# 

**Published:** 1899-11-11

**Authors:** 


					THE HOSPITAL. "The standard of hieh-st purity."?Lanret. November 11th, 1899,
CADBURY'S nf ? CADBURY'S
* \m. )Mk M _ fiOROA
COCOA m m m COCOA
ABSOLUTELY PURE. ^ NO DRU0* OR ALKALIES USED
No. 688. Vol. XXVII. Saturday,
Nov. 11th, 1899.
j-SuhscdpttonTerms.j pRICE TWOPENCE.

				

